# Epidemiology of invasive candidiasis in a surgical intensive care unit: an observational study

**DOI:** 10.1186/s13104-015-1458-4

**Published:** 2015-09-29

**Authors:** Gerardo Aguilar, Carlos Delgado, Isabel Corrales, Ana Izquierdo, Estefanía Gracia, Tania Moreno, Esther Romero, Carlos Ferrando, José A. Carbonell, Rafael Borrás, David Navarro, F. Javier Belda

**Affiliations:** Surgical Intensive Care Unit, Department of Anesthesiology and Intensive Care, Hospital Clínico Universitario de Valencia, Avenida Blasco Ibáñez 17, 46010 Valencia, Spain; Department of Microbiology, Hospital Clínico Universitario de Valencia, Avenida Blasco Ibáñez 17, 46010 Valencia, Spain; School of Medicine, University of Valencia, Avenida Blasco Ibáñez 15, 46010 Valencia, Spain

**Keywords:** Intensive care unit, Invasive candidiasis, Candidemia, Antifungal susceptibility

## Abstract

**Background:**

Invasive candidiasis (IC) is a frequent and life-threatening infection in critically ill patients. The aim of this study was to evaluate the epidemiology of IC and the antifungal susceptibility of etiological agents in patients admitted to our surgical intensive care unit (SICU) in Spain.

**Methods:**

We designed a prospective, observational, single center, population-based study in a SICU. We included all consecutive adult patients (≥18 years old) who had documented IC, either on admission or during their stay, between January 2012 and December 2013.

**Results:**

There were a total of 22 episodes of IC in the 1149 patients admitted during the 24-month study. The overall IC incidence was 19.1 cases per 1000 admissions. Thirteen cases of IC (59.1 %) were intra-abdominal candidiasis (IAC) and 9 (40.9 %) were candidemias. All cases of IAC were patients with secondary peritonitis and severe sepsis or septic shock. The overall crude mortality rate was 13.6 %; while, it was 33 % in patients with candidemia. All patients with IAC survived, including one patient with concomitant candidemia. The most common species causing IC was *Candida albicans* (13; 59.1 %) followed by *Candida parapsilosis* (5; 22.7 %), and *Candida glabrata* (2; 9.1 %). There was also one case each (4.5 %) of *Candida krusei* and *Candida tropicalis*. Thus, the ratio of non-*C. albicans* (9) to *C. albicans* (13) was 1:1.4. There was resistance to fluconazole and itraconazole in 13.6 % of cases. Resistance to other antifungals was uncommon.

**Conclusions:**

*Candida parapsilosis* was the second most common species after *C. albicans*, indicating the high prevalence of non-*C. albicans* species in the SICU. Resistance to azoles, particularly fluconazole, should be considered when starting an empirical treatment. Although IAC is a very frequent form of IC in critically ill surgical patients, prompt antifungal therapy and adequate source control appears to lead to a good outcome. However, our results are closely related to our ICU and any generalization must be taken with caution. Therefore, further investigations are needed.

## Background

Approximately 15 % of health-care associated infections are caused by fungi and *Candida* accounts for 70–90 % of all invasive infections. *Candida* spp. are included in the 10 most common microorganisms causing bloodstream infections (BSI). North American and European studies showed that yeasts belonging to genus *Candida* ranged from the fourth to tenth most common cause of BSI [1]. On the other hand, 30–40 % of episodes of recurrent gastrointestinal tract perforation or acute necrotizing pancreatitis are complicated by intra-abdominal candidiasis (IAC) [2]. However, the diagnosis of non-candidemic invasive candidiasis (IC) remains elusive in the majority of patients. In fact, neither the guidelines from the Infectious Diseases Society of America [3] nor the European consensus [4] provided any clarification on IAC; while, epidemiological data over the last decades have shown than non-candidemic IC, which is mostly peritonitis, is a frequent and life-threatening complication in critically ill surgical patients [5]. Due to the poor outcome associated with IC in critically ill patients, an understanding of local epidemiologic trends and the antifungal susceptibility of etiological agents is crucial. The main goal of this study was to evaluate the epidemiology of IC in our surgical intensive care unit (SICU). Additionally, we evaluated in vitro susceptibility of isolates and outcome of IC in our SICU during a 2-year period.

## Methods

We designed a prospective, observational, single center study between January 2012 and December 2013. All consecutive adult patients (≥18 years old) who had documented IC, either on admission or during their stay, were enrolled. The local ethics committee (*Instituto de Investigación Sanitaria, INCLIVA*) approved the protocol. Informed consent was obtained from patients or their representative.

The definition of IC was the recovery of yeast from blood culture or other normally sterile site. Candidemia was considered to be catheter-related if a catheter tip culture yielded the same yeast isolated in the bloodstream. *Candida* colonization was considered unifocal when *Candida* species were isolated from one non-steril site and considered multifocal when *Candida* species were simultaneously isolated from at least two different non-steril sites, even if two different *Candida* species were isolated [6]. Corticosteroid treatment was defined as exposure to a 10 mg/day prednisone equivalent for ≥30 days. Adequate source control was defined as removal of any preexisting central vein catheters or documented surgical or radiologic procedures to drain abscesses or other fluid collections thought to be the source of *Candida* infection within 24 h of the onset of infection. The severity and organ dysfunction at the IC diagnosis were computed by the sequential organ failure assessment (SOFA) score [7]. Antifungal treatment was considered adequate if the recommended dose of an antifungal drug was administered within 48 h after sample collection for a susceptible *Candida* isolate [8], according to the species-specific clinical breakpoint suggested by the Clinical Laboratory Standards Institute (CLSI) subcommittee [9]. The outcome variables were early (≤7 days) and late (8–30 days) mortality.

### Laboratory procedures

Blood specimens and non-centrifuged peritoneal fluids obtained during surgery (one specimen/patient) were directly inoculated into two bottles of BACTEC media (PLUS aerobic/F and PLUS anaerobic/F; BD Diagnostics, Sparks, MD, USA), which were incubated for a maximum of 7 days, and analyzed using the automated continuous blood culture monitoring BACTEC FX system (BD Diagnostics). The broth was then sub-cultured on Sabouraud Dextrose Agar with Chloramphenicol (SC) when yeast growth was observed. For colonization purposes, pharyngeal and anal exudates were inoculated in Brain Heart Infusion Broth incubated at 37 °C for 24 or 48 h, and then sub-cultured on SC. Urine specimens were directly streaked onto SC. Cultured yeasts were identified based on the macro-microscopic features of the culture, germ tube test, and biochemical tests (VITEK^®^ 2 system bioMérieux, Inc. Hazelwood, MO, USA or API^®^/ID 32C bioMérieux, Marcy l’Etoile, France).

Antifungal susceptibility tests were performed using Sensititre Yeast One^®^ (TREK Diagnostic Systems Ltd.). The minimum inhibitory concentration (MIC) results for all antifungals, except for amphotericin B, were interpreted by taking into account the species-specific clinical breakpoint suggested by the CLSI [9]. In accordance with the literature data, a breakpoint ≤1 μg/ml was selected for amphotericin B to define the isolates as sensitive (S).

### Statistical analysis

Continuous normally distributed data are expressed as the mean and standard deviation (SD) and compared using unpaired Student’s t test. Non-normally distributed data are expressed as the median and interquartile ranges (IQR) and compared using the Mann–Whitney U test. Categorical data are expressed as the number and percentage and compared using *χ*^2^ or the Fisher’s exact tests. In all comparisons, a p < 0.05 was considered to be statistically significant. Data analysis was performed using the *Statistical Package for the Social Sciences* software.

## Results

Baseline characteristics of the patients are shown in Table 1. Among 1149 patients consecutively admitted to our ICU during the 24-month survey, 22 patients with IC were identified. The overall IC incidence was 19.1 cases per 1000 admissions. The most common predisposing factors for IC, present at the time of diagnosis, were central venous catheter (CVC) and urinary catheter presence (100 %), followed by antibiotic therapy (95.4 %), mechanical ventilation (90.9 %), ICU stay ≥7 days (68.2 %), and total parenteral nutrition (63.6 %). The median age of the patients was 66 (53.7–74.2) years, with 72.7 % males and 27.3 % females. The admitting diagnosis was surgical in 90.9 % (70 % intra-abdominal) and medical in 9.1 % of cases. The median SOFA score at enrollment was 5 (3–6.2). In 2 cases (9.1 %), IC was already present at ICU admission, while it occurred during the ICU stay in 20 cases (90.9 %), with a median onset time of 20 days (5–37.5).

**Table 1:** Baseline characteristics of patients with invasive candidiasis (IC)

Characteristics	Yeast infection, n = 22
Age (years), median (IQR)	66 (53.7–74.2)
Male sex, *n* (%)	16 (72.7)
Comorbidities, n (*%*)	21 (95.4)
Chronic renal failure, *n* (%)	3 (13.6)
Diabetes mellitus, *n* (%)	7 (31.8)
Solid tumor, *n* (%)	7 (31.8)
Steroid therapy, *n* (%)	0 (0)
Parenteral nutrition, *n* (%)	14 (63.6)
Mechanical ventilation, *n* (%)	20 (90.9)
Urinary catheter, n (%)	22 (100)
Central venous catheter (CVC), *n* (%)	22 (100)
CVC removed, *n* (%)	22 (100)
Yeast positivity in removed CVC, *n* (%)	2 (9.1)
Previous hospitalization, *n* (%)	16 (72.7)
Pre-IC diagnosis LOS (days), median (IQR)	20 (5–37.5)
LOS >7 days, *n* (%)	15 (68.2)
Admission typology	–
Surgical pathology, *n* (%)	20 (90.9)
Medical pathology, *n* (%)	2 (9.1)
Trauma, *n* (%)	0 (0)
Abdominal surgery, *n* (%)	14 (63.6)
SOFA score^a^ on IC diagnosis, median (IQR)	5 (3–6.2)
Empirical therapy duration (days), median (IQR)	10 (5–16.5)
Previous antibiotic therapy, *n* (%)	21 (95.4)
Concomitant bacterial infection, *n* (%)	20 (90.9)
Concomitant bacteremia, *n* (%)	9 (40.9)
Fungemia, *n* (%)	9 (40.9)
Fungemia duration (days), median (IQR)	7 (9.5–14.5)
Previous fungal invasive infection, *n* (%)	2 (9.1)
*C. albicans* species, *n* (%)	13 (59.1)
Fungal multifocal colonization, *n* (%)	8 (36.4)
Colonization and infection species coincidence, *n* (%)	6 (75)
Mortality (global)*, n* (%)	3 (13.6)
Early mortality (≤7 days), *n* (%)	2 (9.1)
Late mortality (>7 days), *n* (%)	1 (4.5)
Mortality in presence of fungemia, *n* (%)	3 (33.3)
Mortality in absence of fungemia, *n* (%)	0 (0)

*CVC* Central venous catheter, *IQR* interquartile range, *LOS* length of stay, *SOFA* sequential organ failure assessment

^a^In the two patients who developed invasive candidiasis prior to ICU admission, their SOFA score was calculated at ICU admission

### Sites of infection and *Candida* species

Thirteen cases of IC (59.1 %) were IAC and nine (40.9 %) were candidemias. All cases of IAC occurred in patients with secondary peritonitis and severe sepsis or septic shock. All samples of peritoneal fluid were obtained surgically. The *Candida* species found are reported in Table 2, 13 cases of IC (59.1 %) were caused by *Candida albicans* (CA) and 9 (40.9 %) by non-*Candida albicans* (nCA) species (ratio nCA:CA was 1:1.4). *Candida parapsilosis* was the most prevalent nCA species (5; 22.7 %), followed by *Candida glabrata* (2; 9.1 %). A multifocal colonization was documented in eight (36.4 %) patients with IC. In 75 % of these cases, there was colonization by the same yeast species isolated from blood or peritoneal fluid. Catheter removal was possible in all patients immediately after the onset of IC, and a tip culture was performed in all cases. Two cases (22.2 %) of candidemia were catheter-related. The duration of candidemia [median (IQR)] was: 7 (9.5–14.5) days (Table 1). In 20 (90.9 %) patients, the infectious process was a mixed infection caused by yeast and bacteria, with Gram-negative bacteria (68 %), especially *Pseudomonas aeruginosa* (32 %), being most common.

**Table 2:** *Candida* species in 22 patients with IC

Species	Isolates, n (%)
*C. albicans*	13 (59.1)
*C. parapsilosis*	5 (22.7)
*C. glabrata*	2 (9.1)
*C. tropicalis*	1 (4.5)
*C. krusei*	1 (4.5)

### Antifungal susceptibility testing

All isolates were tested for in vitro antifungal susceptibility according to the CLSI breakpoints [9]. The results of antifungal resistance are shown in the Table 3. All isolates were susceptible to amphotericin B, voriconazole, caspofungin, and posaconazole; while, 13.6 % of isolates were resistant to fluconazole (*C. albicans*, 1; *C. glabrata*, 1; *Candida krusei*, 1) and itraconazole (*C. albicans*, 2; *C. krusei*, 1). In one patient, *C. parapsilosis* was resistant to anidulafungin and micafungin with a MIC of 4 mg/L for both echinocandins.

**Table 3:** Antifungal resistances in 22 *Candida* isolates from patients with IC

Antifungal	Resistance, n (%)
5-fluorocytosine	1 (4.5)
Amphotericin B	0 (0)
Fluconazole	3 (13.6)
Voriconazole	0 (0)
Caspofungin	0 (0)
Posaconazole	0 (0)
Micafungin	1 (4.5)
Anidulafungin	1 (4.5)
Itraconazole	3 (13.6)

### Treatment

No patient was on antifungal prophylaxis at the time of IC diagnosis. Empirical therapy was started in 100 % of patients within 24 h of the onset of symptoms and the duration was 10 (5.0–16.5) days. The drugs empirically prescribed were echinocandins (86.4 %, 19 patients), followed by fluconazole (13.6 %, 3 patients). In all cases, empirical therapy was confirmed as the target therapy. Eight patients (36.4 %) underwent de-escalation with fluconazole.

### Patient outcome

Overall, the crude mortality rate was 13.6 %; while, it was 33.3 % in patients with candidemia (two patients with *C. albicans* and one patient with *C.parapsilosis*). All patients with IAC survived, including one patient with concomitant candidemia. Early and late mortality were 9.1 and 4.5 %, respectively. Data on the severity, organ dysfunction, lenght of stay in the SICU and mortality according to the type of IC are shown in the Table 4.

**Table 4:** Candidemia and intra-abdominal candidiasis (IAC) group characteristics

Characteristics	Candidemia, n = 9	IAC, n = 13
SOFA score on diagnosis, median (IQR)	5.00 (4.5–7)	4 (3–6)
Length of stay in the SICU, days, median (IQR)	26.00 (6–40.5)	10 (5–31.5)
Mortality*, n* (%)	3 (33)	0 (0)
Early mortality (≤7 days), *n* (%)	2 (22.2)	0 (0)
Late mortality (>7 days), *n* (%)	1 (11.1)	0 (0)

*IQR* interquartile range, *SOFA* sequential organ failure assessment

In all comparisons between groups there were no statistically significant differences (*p* > 0.05)

## Discussion

We have reported an overall IC incidence of 19.1 cases per 1000 admissions. This finding is higher than the data reported for Northern Europe (6.7–7.4 cases per 1000 admissions) [10, 11] or Italy (16.5 cases per 1000 admissions) [12] and lower than those reported in other European countries (35.7–54 cases per 1000 admissions) [13, 14]. We found that *C. albicans* was the leading agent (13 cases; 59.1 %), followed by *C. parapsilosis* (4 cases; 22.7 %) then *C. glabrata* (2 cases; 9.1 %). Although *C. albicans* is still regarded as the most common species [3], there has been an increasing incidence of nCA infections with *C. glabrata* and *C. parapsilosis* [15–18]. In our country, a recent multicenter study about candidemias demonstrated an increase in nCA infections in the ICU [19]. Our data confirm this increase and *C. parapsilosis* was the most common among the nCA species.

It is noteworthy that the resistance to itraconazole and, especially, fluconazole was 13.6 %. However, resistance to echinocandins was low in our patients (Table 3). Only one patient (who survived) with *C. parapsilosis* was resistant to anidulafungin and micafungin, according to the breakpoints used in our study [9], with a MIC of 4 mg/L for both antifungals. Nonetheless, in this case, anidulafungin was used empirically, improving the signs and symptoms of sepsis immediately after the onset of the treatment. There was clinical and microbiological resolution in the following days and a successful outcome. The MIC values published recently [20] were higher than the MICs used in our study [9], and this might explain the patient’s good clinical response to treatment.

The most frequent IC in our patients was IAC (59.1 %). Previous studies have demonstrated that fungal infections adversely affect the outcome of patients with peritonitis. In fact, isolation of *Candida* from peritoneal fluid has been associated with high mortality [21, 22]. In IAC, as in candidemias, the delay in the initiation of antifungal therapy is a major determinant of clinical outcome [23, 24]. It is striking that, in our study, all patients with IAC survived. It is likely that the prompt (within 24 h of IC onset) and appropriate antifungal therapy and adequate source control could explain these outcomes. There is sufficient evidence in the literature to support the strategy of early administration of antifungal therapy and adequate source control in patients with IC [8, 25].

In the subgroup of patients with candidemia (n = 9), the CVC was removed in all cases, but the candidemia was catheter-related in only two cases (22.2 %). The mortality rate in candidemic patients was 33.3 % (3 patients). In these patients, candidemia (2 *C. albicans*, 1 *C. parapsilosis*) was considered primary, with an unknown origin. In fact, primary candidemia was recently identified as an independent risk factor for mortality in candidemic patients [19].

There are some limitations in our study. First, this is a single center study of a critically ill surgical patient population and our epidemiology cannot be extrapolated to all settings. The second limitation is that the frequency of colonization by *Candida* species was probably underestimated because surveillance cultures were not systematically screened. Finally, due to the small sample size (n = 22) direct comparisons between the two groups of IC (IAC and candidemia) could not be reliably performed.

## Conclusions

Our study confirmed the high prevalence of nCA species in critically ill surgical patients and *C. parapsilosis* as the most common species after *C. albicans*. Resistance to azoles, particularly fluconazole, should be considered when starting an empirical treatment. The most common IC was IAC in this study of critically ill surgical patients. Although the isolation of *Candida* spp. in peritoneal specimens of nosocomial peritonitis has been suggested as an independent risk factor of mortality, all patients with IAC survived in this study. The prompt antifungal therapy and adequate source control in our patients likely explains their good outcome. However, our results just reflect the state of the matter at our ICU and naturally can not be extrapolated to other ICUs. Therefore, further investigation is necessary.

## Key points

- In surgical intensive care patients, the prevalence of non-*Candida albicans* species has increased in the last few years.
- Resistance to fluconazole should be considered when initiating an empirical antifungal treatment in critical care surgical patients.
- Intra-abdominal candidiasis is a very frequent form of invasive candidiasis in the critical care surgical setting.
- Prompt antifungal therapy and adequate source control might explain the good outcome of our patients with intra-abdominal candidiasis.
- Additional larger multicenter studies are needed to confirm our findings.

## Abbreviations

BSI: bloodstream infections
CA: *Candida albicans*
CLSI: Clinical Laboratory Standards Institute
CVC: central venous catheter
IAC: intra-abdominal candidiasis
IC: invasive candidiasis
IQR: interquartile range
MIC: minimum inhibitory concentration
nCA: *non*-*Candida albicans*
SC: Sabouraud Dextrose Agar with Chloramphenicol
SD: standard deviation
SICU: surgical intensive care unit
SOFA: sequential organ failure assessment
